# Development of potato (Solanum tuberosum L.) plants with inactivated StPain-1 gene using CRISPR/Cas9

**DOI:** 10.18699/vjgb-26-61

**Published:** 2026-07

**Authors:** V.D. Karlov, M.K. Volkov, A.D. Antipov, Yu.S. Monahova, A.S. Trofimov, L.N. Konovalova, A.V. Babakov, R.A. Komakhin, V.V. Taranov

**Affiliations:** All-Russia Research Institute of Agricultural Biotechnology, Moscow, Russia; All-Russia Research Institute of Agricultural Biotechnology, Moscow, Russia; All-Russia Research Institute of Agricultural Biotechnology, Moscow, Russia; All-Russia Research Institute of Agricultural Biotechnology, Moscow, Russia; All-Russia Research Institute of Agricultural Biotechnology, Moscow, Russia; All-Russia Research Institute of Agricultural Biotechnology, Moscow, Russia; All-Russia Research Institute of Agricultural Biotechnology, Moscow, Russia; All-Russia Research Institute of Agricultural Biotechnology, Moscow, Russia; All-Russia Research Institute of Agricultural Biotechnology, Moscow, Russia

**Keywords:** vacuolar invertase, cold-induced sweetening, sucrose, glucose, fructose, potato, CRISPR/Cas9, guide RNA, вакуолярная инвертаза, холодовое осахаривание, сахароза, глюкоза, фруктоза, картофель, CRISPR/Cas9, гидовая РНК

## Abstract

Storage of potato tubers intended for further processing is complicated by their cold-induced sweetening (CIS). Enzymatic hydrolysis of sucrose into glucose and fructose occurs at low temperatures. The resulting high hexose content adversely affects the quality of processed potato products such as chips and fries and promotes the formation of acrylamide, which is a neurotoxin and carcinogen. During CIS, sucrose hydrolysis is catalyzed by vacuolar invertase encoded by the StPain-1 gene. Previous studies have shown that suppression of the enzyme activity confers potato resistance to CIS without reducing the nutritional value of tubers. In this study, CRISPR/Cas9 technology was used to generate Solanum tuberosum L. cv. Fritella plants with a knockout of StPain-1. Two binary vectors based on pKSE401 were constructed (Vector A and Vector B), each carrying two gRNAs targeting exon 1 (sgRNA-P1.A or sgRNA-P1.B) and exon 3 (sgRNA-P3.A or sgRNA- P3.B). Editing efficiency with each gRNA was evaluated through next-generation sequencing (NGS). Transformation with Vector A produced 48 transformants, 22 of which carried knockouts in all StPain-1 alleles. Transformation with Vector B yielded 26 transformants, including 10 plants with complete StPain-1 knockout. Chips made from tubers of nine edited Fritella plants demonstrated reduced vacuolar invertase activity: chips from StPain-1 knockout lines were lighter compared to the non-edited control sample. Quantitative assessment of glucose, fructose, and sucrose levels, as well as StPain-1 mRNA expression in tubers of four selected transformants (two per vector), confirmed enzyme inactivation. The resulting plants exhibit increased resistance to cold-induced sweetening and can be used as a promising source of nonfunctional StPain-1 alleles for breeding new potato varieties.

## Abstract (ru)

Холодовое хранение клубней картофеля для последующей переработки осложнено развитием холодового осахаривания. При пониженной температуре происходит реакция ферментативного гидролиза сахарозы на глюкозу и фруктозу. Как следствие, высокое содержание гексоз негативно сказывается на качестве продуктов переработки картофеля – чипсов и фри, а также способствует образованию нейротоксина и канцерогена акриламида. Гидролиз сахарозы в процессе холодового осахаривания катализирует вакуолярная инвертаза, кодируемая геном StPain-1. Ранее было показано, что подавление активности фермента придает картофелю устойчивость к холодовому осахариванию, не снижая при этом питательных качеств клубней. В настоящей работе с помощью CRISPR/Cas9 были созданы растения культурного картофеля Solanum tuberosum L. сорта Фрителла с нокаутом гена StPain-1. Для редактирования целевого гена были собраны две генетические конструкции на основе вектора pKSE401 (Вектор A и Вектор B), каждая из которых несла две гидовые РНК: на участки в первом (sgRNA-P1.A и sgRNA-P1.B) и третьем (sgRNA-P3.A и sgRNA-P3.B) экзонах. Эффективность редактирования каждой gRNA была охарактеризована с применением высокопроизводительного секвенирования. С конструкцией Вектор A получено 48 трансформантов, среди которых 22 содержали нокаут всех аллелей гена StPain-1; с конструкцией Вектор B – 26 трансформантов, из них 10 были с нокаутом. Путем обжарки чипсов из клубней девяти отредактированных растений картофеля сорта Фрителла было наглядно продемонстрировано снижение активности вакуолярной инвертазы. Чипсы из образцов с нокаутом гена StPain-1 имели более светлую окраску, чем чипсы из контрольного образца без редактирования. По результатам проведенной работы отобрано четыре растения с нокаутом гена StPain-1, по два для каждой конструкции, для которых количественная оценка глюкозы, фруктозы и сахарозы, а также измерение уровня мРНК подтвердили инактивацию фермента. Полученные растения обладают повышенной устойчивостью к холодовому осахариванию и являются перспективными источниками нефункциональных аллелей гена StPain-1 для селекции новых сортов картофеля.

## Introduction

Potato (Solanum tuberosum L.) is one of the world’s most important food crops. It ranks third among crops consumed worldwide and second in Russia (Osipov, Zeldner, 2023). In countries with temperate climate, potatoes are harvested once a year. In order to preserve commercial qualities, harvested tubers are stored at temperatures of 4–10 °C (Wustman, Struik, 2007). At these storage temperatures, the process called cold-induced sweetening (CIS) occurs in the tubers. It involves excessive accumulation of glucose and fructose (Zrenner et al., 1996). Tubers having undergone CIS are unsuitable for further processing into chips or fries, as they turn dark and bitter (Murata, 2021).

Vacuolar invertase (β-fructofuranosidase; EC 3.2.1.26) plays a key role in CIS (Davies et al., 1989; Richardson et al., 1990; Blenkinsop et al., 2003). It catalyzes the hydrolysis of sucrose into hexoses: glucose and fructose (Zrenner et al., 1996; Zhu et al., 2024). During hightemperature processing, the Maillard reaction occurs, whereby the hexoses interact with the amino groups of proteins and free amino acids to produce melanoidin pigments. Another product of this reaction is acrylamide, a neurotoxin and carcinogen that poses a hazard to human health (Tareke et al., 2002; Siaw et al., 2018).

The glucose and fructose contents largely determine the color, taste, and safety of roasted potato products (Siaw et al., 2018). Use of tubers with hexose content greater than 1 mg per 1 g of fresh weight (mg/g FTW) is not recommended due to high accumulation of acrylamide in the final product (Biedermann-Brem et al., 2003).

Several experimental approaches have been developed to suppress the enzymatic activity of vacuolar invertase in order to reduce hexose levels and increase the resistance of potato tubers to CIS. These approaches include the overexpression of genes for natural vacuolar invertase inhibitors (Greiner et al., 1999; Mckenzie et al., 2013), the suppression of the endogenous StPain-1 gene expression via RNA silencing (Zrenner et al., 1996; Zhang et al., 2008; Bhaskar et al., 2010; Wu et al., 2011; Zhu et al., 2014; Toufiq et al., 2025), and the inactivation of the StPain-1 gene using genome editing techniques (Clasen et al., 2016; Yasmeen et al., 2022; Ly et al., 2023; Teper‐ Bamnolker et al., 2023; Shumbe et al., 2024; Egorova et al., 2025; Massa et al., 2025).

Genome editing appears to be the most promising method for raising potato varieties resistant to CIS, as it enables the creation of new, nonfunctional variants of the StPain-1 gene. These nonfunctional StPain-1 alleles can then be transferred to new potato varieties through crossbreeding. Furthermore, as demonstrated in (Yasmeen et al., 2022), it is sufficient to inactivate only part of the alleles to effectively reduce vacuolar invertase activity.

Previously, traditional breeding methods were used to develop potato varieties intended for processing into chips and fries, such as Queen Anne, Lady Claire, Barin, Vympel, and others. Tubers of the aforementioned potato varieties accumulate less glucose and fructose during cold storage. However, hexose content is sufficient to reduce the quality of the final product (Kulakova et al., 2022; Bruno et al., 2025). The growing demand for potato products means a need for developing new varieties that would be more resistant to CIS.

Although several potato genotypes resistant to CIS have been created based on the Atlantic, Desiree, and Symphony varieties using genome editing techniques, legislative restrictions in a number of countries prevent the free use of such plants for industrial purposes due to the presence of a transgene (Ly et al., 2023; Teper‐ Bamnolker et al., 2023; Egorova et al., 2025; Massa et al., 2025).

The aim of this study was to knock out the vacuolar invertase gene in Fritella potato in order to obtain a plant source of nonfunctional StPain-1 alleles for the breeding of new varieties (Simakov et al., 2017). Fritella variety was chosen due to high tuber suitability for making French fries and chips. The plants developed in this study show promise as donors for the selection of new nontransgenic potato varieties with increased resistance to CIS.

## Materials and methods

Plant material. Aseptic tetraploid potato plants (S. tuberosum L.) of the Fritella cv. (provided by the A.G. Lorch Research Institute of Potato Farming, Moscow) were cultivated on MS medium (Murashige, Skoog, 1962) at 20–22 °C, with the 16-hour light/8-hour dark schedule, and a photosynthetic photon flux density (PPFD) of 80 μmol m−2 s−1. The absence of viral contamination in the aseptic plants was confirmed by real-time PCR analysis with a commercially available kit “PHYTOSCREEN Potato Virus X, Y, M, L, S, A, PSTVd-RT” in expert molecular study No. 57/2023 dated July 26, 2023 by Syntol LLC (Russia). Agrobacterium-mediated transformation of aseptic leaf explants was performed according to a previously published method (Vetchinkina et al., 2016). Potato plants were grown in vessels in a greenhouse at 20–21 °C, with the 16-hour light/8-hour dark schedule and PPFD of 150 μmol m−2 s−1. Tubers were stored in the dark at 4 °C.

Agrobacterium strain. Agrobacterium tumefaciens bacteria of AGL0 strain were used to transform potato explants.

gRNA selection and cloning. Genomic DNA was extracted from Fritella potato leaves using cetyl trimethylammonium bromide (CTAB). Coding sequences of the first and third exons of the StPain-1 gene were analyzed using CRISPOR (https://crispor.org) to choose gRNAs (Concordet, Haeussler, 2018). Four sgRNAs were used in the study: two gRNAs targeting exon 1 (sgRNA-P1.A and sgRNA-P1.B), and the other two targeting exon 3 (sgRNA-P3.A and sgRNA-P3.B). The sequence of sgRNA-P3.A was taken from (Yasmeen et al., 2022). gRNAs were cloned in pairs into the pKSE401 binary vector for genome editing according to the recommendations (Xing et al., 2014). This resulted in the creation of Vector A (sgRNA-P1.A and sgRNA-P3.A) and Vector B (sgRNA-P1.B and sgRNA-P3.B).

Genotyping of transgenic plants. Genomic DNA was extracted from leaves of reproductive T0 generation plants using CTAB. Regions of exons 1 and 3of the StPain-1 gene were amplified using the oligonucleotides listed in Supplementary Table S11.

Supplementary Materials are available in the online version of the paper: https://vavilov.elpub.ru/jour/manager/files/Suppl_Karlov_Engl_30_4.xlsx


For PCR, a mixture containing 1× Taq polymerase buffer, 0.2 mM dNTP, 0.5 μM of each primer, 1.25 U of Taq DNA polymerase (Eurogen, Moscow), and 20 ng of genomic DNA was prepared and adjusted to a final volume of 25 μL. PCR was performed under the following conditions: predenaturation at 94 °C for 2 min followed by 30 cycles of 10 s at 94 °C, 15 s at 58–62 °C, 40 s at 72 °C, and then the final elongation step at 72 °C for 5 min. The amplicons were resolved in 1.5 % agarose gel and purified with a ColGen kit (Syntol, Moscow). The amplicon sequencing was performed on the Illumina MiSeq platform in the Biotechnology Shared Access Center at the All-Russia Research Institute of Agricultural Biotechnology. Sequencing quality was assessed with the MultiQC tool ver. 1.22.2 (Ewels et al., 2016). Editing efficiencies for each target region in each plant were determined as a percentage of the number of reads containing mutations relative to the total number of reads, using CRISPResso2 ver. 2.3.1 software (Clement et al., 2019).

Sugar content assessment. Tuber tissue (100 mg) was homogenized with a pestle in 500 μL of water. The volume of the homogenate was adjusted to 1,000 μL with ddH2O and mixed by vortexing. After centrifugation at 13,000g for 2 min the debris was removed. To measure the sugar content, 100 μL of the extract was taken. The glucose, fructose, and sucrose contents were quantified with commercially available Enzytec Liquid D-Glucose/D-Fructose and Enzytec Liquid Sucrose/ D-Glucose kits (R-Biopharm, Germany), following the manufacturer’s recommendations. The glucose, fructose, and sucrose contents are given in mg/g FTW.

Starch content assessment. A piece of tuber (50 mg) was homogenized with a pestle in 500 μL of a 4:1 mixture of dimethyl sulfoxide (DMSO) and hydrochloric acid (HCl). The suspension volume was adjusted to 1.25 mL using the same mixture, mixed by vortexing, and incubated at 60 °C for 30 min. The suspension was diluted with 2.5 mL of ddH2O and titrated with 5 M NaOH until pH 4–5. The mixture was centrifuged at 13,000g for 2 min, and 100 μL of the supernatant was collected for analysis. Quantitative assessment was performed using a commercially available Enzytec Liquid Starch kit (R-Biopharm, Germany), following the manufacturer’s recommendations. The starch content is given in mg/g FTW).

RNA extraction. To extract RNA, 100 mg of tuber tissue was crushed with a pestle in a mortar containing liquid nitrogen. RNA was then extracted by the column method using a SKYprep RNA Pure Plant Plus Kit (SkyGen, Russia). The RNA quality was checked by electrophoresis in 1.5 % agarose gel.

Reverse transcription. Reverse transcription was performed using MMLV reverse transcriptase (Evrogen, Russia). To perform the reaction, a mixture of 1× First strand buffer, 0.2 mM dNTP mix, 2 uM oligo(dT)15 primer, 1 mg of isolated RNA, 2 mM DTT, and 100 U of MMLV-RT was prepared with a final volume of 20 μL according to the manufacturer’s recommendations. The reaction was carried out for 60 min at 37 °C, followed by 10-min incubation at 70 °C. The synthesized cDNA was then used as a template for quantitative PCR.

Quantitative PCR analysis. The reaction mixture was prepared using 5× qPCRmix-HS SYBR (Evrogen, Russia). The oligonucleotides used are listed in Table S1. The reaction was carried out under the following conditions: 3 min at 95 °C, followed by 50 cycles of 10 s at 95 °C, 30 s at 60 °C, and 30 s at 72 °C. The Tubulin and EF-1a genes were used as reference sequences (Table S1) (Nicot et al., 2005; Zhu et al., 2014). The relative expression of the StPain-1 gene was calculated using the 2−ΔΔCt method (Vandesompele et al., 2002).

Frying chips from tubers. Tubers harvested from the T0 vegetative generation after 2 months of storage at 4 °C were cut into ~1–2 mm slices and fried in vegetable oil at 170 °C for 2 min.

Statistical analysis. All experiments were performed in triplicate. The resulting data were processed by ANOVA. The studied samples were compared with the control group using the Dunnett test. Mean values and their standard deviations were calculated to construct glucose, fructose, sucrose and starch content and relative expression charts. All the calculations were performed in GraphPad Prism 8.

## Results

**Choice of guide RNAs to introduce knockout mutations into the vacuolar invertase gene StPain-1**

**Fig. 1.**
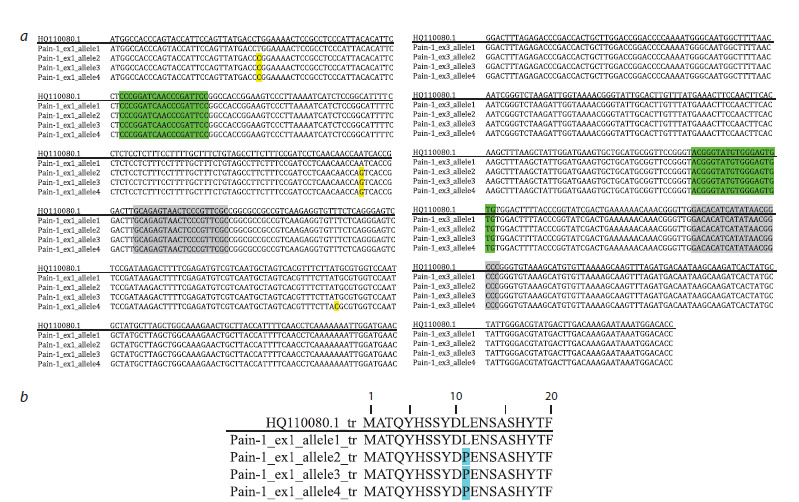
The similarity in the StPain-1 gene regions across four alleles. a – alignment of the nucleotide sequences of exon 1 and fragment of exon 3 of four identified StPain-1 gene alleles to the reference sequence (Madeira et al., 2024). Polymorphisms are shaded yellow. Editing sites are shaded green for Vector A and gray for Vector B; b – translation of the first 20 codons of the reference StPain-1 sequence and the sequences of the four alleles found in the genome of the Fritella potato cultivar. Polymorphisms in the amino acid sequences of the alleles are shaded blue.

Vacuolar invertase gene StPain-1 comprises seven exons and six introns (Abbas et al., 2022). Exons 1 and 3 were selected as targets for editing since introducing mutations into an upstream part of a gene increases knockout probability. Amplicons of exons 1 and 3 of the StPain-1 gene obtained from the genomic DNA of the Fritella cv. were sequenced so as to choose gRNAs afterwards. Alignment of the nucleotide sequences of the first exon amplicons to the reference sequence of the StPain-1 gene (NCBI: HQ110080.1) revealed several single nucleotide polymorphisms at the 32nd, 174th, and 288th positions from the start codon. Among these, the substitution of C for T at the 32nd position (c.32T>C) resulted in the amino acid substitution Leu>Pro in three of the four alleles (Fig. 1a, b). The nucleotide sequences of the exon 3 fragment in all alleles were identical to the reference sequence (Fig. 1a).

**Fig. 2.**
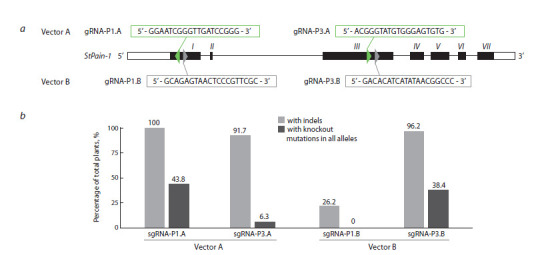
Efficiency of introducing mutations into the vacuolar invertase gene StPain-1 of Fritella plants using different guide RNAs. a – the exon-intron structure of the StPain-1 gene and the location of the guide RNAs for Vector A (highlighted in green) and Vector B (highlighted in gray). Arrows indicate the directions of the guide RNAs; b – percentages of plants with indel mutations (shown in gray) and with knockout mutations in all alleles (shown in black) of the StPain-1 gene among transformants. For Vector A, n = 48 transformants; for Vector B, n = 26 transformants.

Four gRNAs were selected to edit the most conserved regions of the vacuolar invertase gene: two gRNAs targeting exon 1 (sgRNA-P1.A and sgRNA-P1.B), and the other two targeting exon 3 (sgRNA-P3.A and sgRNA-P3.B) (Fig. 1a and 2a). The sequences of three gRNAs (sgRNA-P1.A, sgRNA-P1.B, and sgRNAP3. B) were selected using the CRISPOR tool, and one guide RNA (sgRNA-P3.A) was taken from an earlier work (Yasmeen et al., 2022). Two pairs of gRNAs were cloned into two genetic constructs, designated Vector A (sgRNA-P1.A and sgRNA-P3.A) and Vector B (sgRNA-P1.B and sgRNA-P3.B) (Fig. 2a). The simultaneous use of two guide RNAs in a single genetic construct was required to increase the probability of gene knockout due to an additive effect in case of low editing efficiency.

**The efficiency of StPain-1 gene editing process depends on the selection of gRNA**

The Agrobacterium-mediated transformation of potato leaf explants with Vector A and with Vector B yielded 48 and 26 regenerants, respectively. The editing efficiency was evaluated using targeted high-throughput sequencing on the Illumina platform. The average number of reads was 1,447, ranging from a minimum of 128 to a maximum of 4,251. Genotyping of the transformants revealed that both genetic constructs introduced indel mutations with high efficiency (Fig. 2b).

The highest mutation efficiency was achieved using the guide RNAs sgRNA-P1.A and sgRNA-P3.B, which created indels in 100 and 96.2 % of transformants, respectively. However, knockout of all StPain-1 alleles was achieved in 43.75 % of transformants using sgRNAP1. A and in 38.4 % of transformants using sgRNA-P3.B. Despite the high efficiency of introducing indels (91.7 % of transformants) with sgRNA-P3.A, knockout of all StPain-1 alleles was detected in only 6.25 % of transformants (3 out of 48). The sgRNA-P1.B gRNA showed the lowest efficiency in introducing indels, which were observed in only 26.9 % of plants (7 out of 26). Also, no transformants with knockout of all StPain-1 alleles were obtained using sgRNA-1.B. The percentages of indels and knockout mutations in transformants as calculated from the NGS results are shown in Tables S2 and S3.

**The diversity of mutant variants depends on the editing site**

**Fig. 3.**
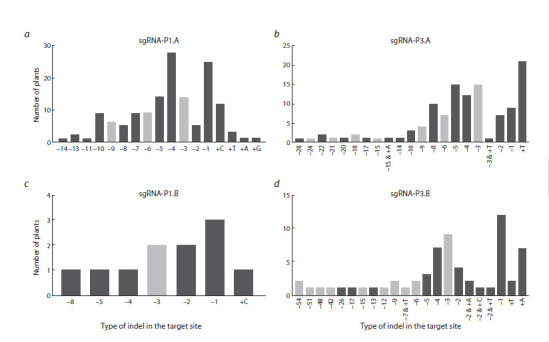
Diversity of indel mutations causing (black bars) and not causing (gray bars) open reading frame shifts in the StPain-1 gene in Fritella transformants created using the Vector A construct with sgRNA-P1.A (a) and sgRNA-P3.A (b) or Vector B with sgRNA-P1.B (c) and sgRNA-P3.B (d) guides

Targeted sequencing on the Illumina platform allowed characterization of the diversity of types of indels obtained with each of the four gRNAs (Fig. 3). The numbers of mutation variants correlated with gRNA efficiency: the most efficient guide RNAs generated a greater diversity of indels. The vast majority of mutations were deletions of 1–10 nucleotides.

StPain-1 gene editing with Vector A resulted in the detection of 17 different indels in exon 1. The most prevalent of these were deletions of 4 nucleotides (in 28 plants) and 1 nucleotide (in 24 plants). The largest deletion included 14 nucleotides. Three indel types did not cause open reading frame shift: deletions of 3, 6, and 9 nucleotides (Fig. 3a). Twenty-one types of indels were found in exon 3. The most common of them were insertions of 1 nucleotide with a thymine base (in 21 plants) and deletions of 5 and 3 nucleotides (in 15 plants each). The largest deletion in exon 3 included 28 nucleotides (Fig. 3b).

Use of the Vector B construct in exon 1 resulted in the detection of only 7 mutation variants: deletions of 8, 5, 4, 3, 2, and 1 nucleotides and an insertion of 1 nucleotide with a cytosine base (Fig. 3c). Exon 3 exhibited the greatest diversity of indels (22 types). The most common was a deletion of 1 nucleotide (12 plants). The largest deletion included 54 nucleotides (two plants). Almost half of the detected indel mutations were deletions of multiples of three nucleotides (Fig. 3d). It can therefore be concluded that a high level of gRNA efficiency is associated with the generation of a wide variety of indel mutations. Furthermore, the diversity of these mutations is determined by the specific editing site. The vast majority of indel mutations are deletions of 1 to 10 nucleotides (see Tables S2–S7 for details).

**Evaluation of plants with StPain-1 gene knockout for resistance to cold-induced sweetening**

For phenotypic evaluation of transformant resistance to CIS by frying chips, tubers were taken from nine plants obtained using Vector A (5 - 2, 11 - 2, 12 - 1, 13 - 2, 26 - 1, 36 - 1, 45 - 1, 49 - 1, 57 - 2) and from five plants obtained using Vector B (16 - 1, 20 - 1, 42 - 2, 50 - 2, 56 - 2). Tubers from transgenic plant 27 - 3 (Vector B) that did not contain indels in any of the StPain-1 alleles were used as a control (see the Table).

**Table 1.**
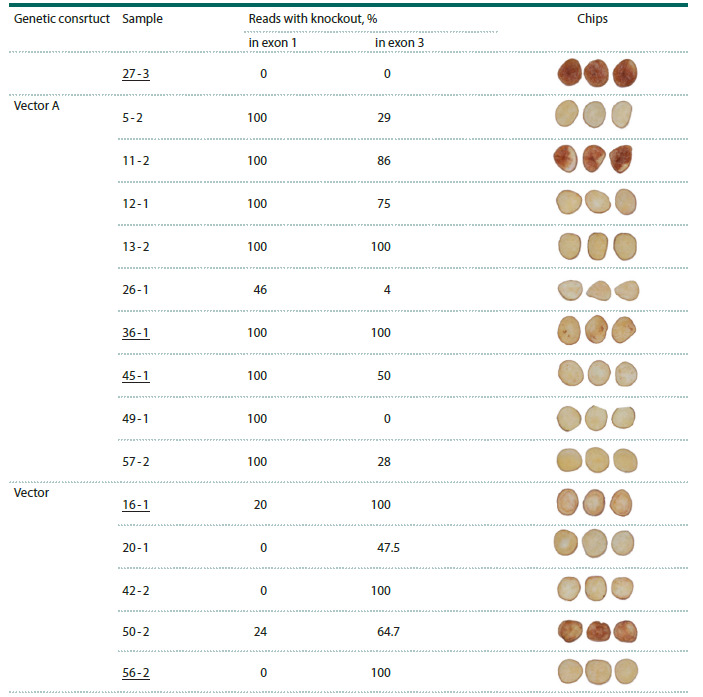
Characteristics of Fritella transformants based on the knockout mutation content in exons 1 and 3 of the StPain-1 gene and the reaction of reducing sugars in tuber slices upon frying Note. Samples taken for the assessment of starch and sugar content, as well as StPain-1 mRNA levels, are underlined.

Chip frying showed that samples with edited vacuolar invertase gene variants were lighter in color than the control sample. Sample 11 - 2 (Vector A) showed a clear discrepancy between the genotype and phenotype: the chips were almost completely dark despite the knockout being confirmed by NGS (see the “Discussion” section for details).

The experiment also included several samples from plants containing both edited and wild-type alleles (26 - 1 – Vector A and 20 - 1, 50 - 2 – Vector B). The color of the chips from sample 50 - 2 was noticeably lighter than that of the control but darker than the knockout samples. Meanwhile, samples 26 - 1 and 20 - 1 had enough inactivated alleles to keep the chips light in color. A similar effect was previously observed by A. Yasmeen et al. (2022), where two of the four StPain-1 alleles were inactivated, ensuring CIS resistance in edited plants (see the “Discussion” section for details). Tubers of four transformants were selected for subsequent measurement of StPain-1 mRNA levels and quantification of sugars and starch: 36 - 1 and 45 - 1 (Vector A), 16 - 1 and 56 - 2 (Vector B).

**Fig. 4.**
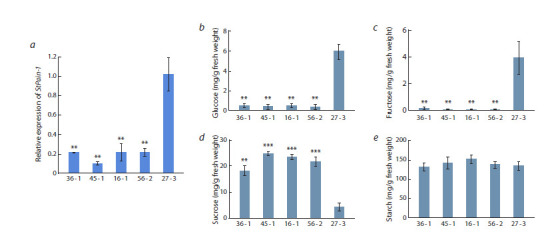
Assessment of carbohydrate contents and levels of StPain-1 mRNA in tubers of some Fritella potato transformants with knockout of all alleles of the StPain-1 gene a – comparison of StPain-1 mRNA levels relative to the reference genes Tubulin and EF-1a in potatoes by real-time PCR. Carbohydrate contents: b – glucose, c – fructose, d – sucrose, e – starch, measured in mg per g of fresh tuber weight. The graphs show the mean values (n = 3) and their standard errors. The significance of the difference calculated using Dunnett’s multiple comparison test is indicated with asterisks (*) P ≤ 0.05; (**) P ≤ 0.01; (***) P ≤ 0.001.

The introduction of indel mutations into the coding sequence of a gene leads to the synthesis of aberrant mRNA, which is destroyed by cellular mRNA quality control systems. When measuring StPain-1 expression in the tubers of selected plants with a knockout of the target gene, a significant decrease in the level of the corresponding mRNA was observed relative to the Tubulin and EF-1a genes (Fig. 4a).

To confirm the inactivation of the vacuolar invertase enzyme, the sugar and starch contents were assayed. The glucose contents in the knockout samples ranged from 0.2 to 0.6 mg/g FTW (Fig. 4b), whereas the fructose content ranged from 0.1 to 0.2 mg/g FTW (Fig. 4c). These values were significantly lower than those observed in the control sample. Conversely, the sucrose content of the StPain-1 knockout samples ranged from 18.1 to 24.5 mg/g FTW, which was significantly higher than in the control sample (Fig. 4d). As expected, no differences were observed between the samples when the starch content in the tubers was measured (Fig. 4e).

This work resulted in the development and characterization of Fritella plants with a knockout of the StPain-1 gene. Glucose and fructose levels in tubers of the obtained plants were below the recommended amount of 1 mg of hexoses per 1 g FTW (Biedermann-Brem et al., 2003). These plants are a promising source of nonfunctional StPain-1 alleles for breeding new potato varieties resista to CIS.

## Discussion

Modern approaches to targeted genome mutagenesis have accelerated the development of new crop varieties significantly. The key objectives in modern breeding programs are to enhance nutritional quality and increase resistance to abiotic and biotic stresses, ensuring more efficient usage of natural resources and reducing economic costs in agriculture. This study focuses on an economically important issue in food industry: CIS of potato tubers, which is caused by vacuolar invertase.

The CRISPR/Cas9 tool was used to inactivate the vacuolar invertase gene StPain-1. Based on the pKSE401 vector (Xing et al., 2014), two constructs containing a pair of guide RNAs each were created: Vector A and Vector B. In each construct, one gRNA targeted exon 1 and the other targeted exon 3. Both genetic constructs demonstrated high efficiency in editing the target gene. According to NGS data, Vector A resulted in indel mutations in at least one allele of the target gene in all 48 regenerants, whereas Vector B produced indel mutations in 25 out of 26 regenerants. Moreover, Vector A knocked out all StPain-1 alleles in 22 out of the 48 regenerants, whereas Vector B, in only 10 out of the 26. The high efficiency of target gene editing was largely ensured by one of the two cloned gRNAs: sgRNA-P1.A for Vector A and sgRNA-P3.B for Vector B.

This work clearly demonstrates the critical importance of choosing right guide RNAs for gene editing in crops. Targeted amplicon sequencing was carried out to compare the efficiency with which four guide RNAs introduced indels. The sgRNA-P1.A, sgRNA-P1.B, and sgRNA-P3.B sequences were chosen for their predicted high efficiency and low potential for side effects, as determined by the CRISPOR and CRISPR-P algorithms (Lei et al., 2014; Concordet, Haeussler, 2018).

The sgRNA-P3.A sequence was taken from the literature (Yasmeen et al., 2022). In practice, however, sgRNA-P1.B exhibited the lowest editing efficiency. It produced indel mutations in only 7 out of 26 plants and not in all alleles. The outcome of plant genome editing depends on various factors, including the delivery method of the editing system, the stability of the gRNACas9 complex, the spatial organization of the genome and the accessibility of the target site to the gRNA-Cas9 complex, the functioning of genome repair systems, etc. In (Egorova et al., 2025), three guide RNAs (gRNA1, gRNA2, and gRNA3) were used within a single construct to edit the StPain-1 gene. Two of these (gRNA1 and gRNA2) targeted sites in the first exon. Upon sequencing the resulting plant sample, it was found that editing with gRNA2 was much more effective than with gRNA1. Conversely, despite its high predicted activity, gRNA3 exhibited the lowest efficiency among all the guide RNAs used in the experiment. Furthermore, the editing efficiency may depend on the cultivar. For example, the StPain-1 gene knockout was more efficient in the Atlantic cv. than in Spunta when the same guide RNAs were employed (Massa et al., 2025).

The CRISPR/Cas9 system is renowned for its high specificity. However, when editing organisms, an offtarget effect often occurs, whereby indel mutations are introduced into non-target regions of the genome with a partially complementary gRNA sequence. These additional mutations can affect the phenotype of the plant. Obviously, sets of off-target sites are different with different guide RNAs. Therefore, plants obtained using different guide RNAs will have different sets of off-target mutations. In this study, the usage of two different genetic constructs enabled us to monitor the potential impact of off-target editing in the assessment of the phenotypes of edited plants.

Editing the StPain-1 gene produced a variety of mutant alleles, the majority of which exhibited deletions of 1, 3, or 4 nucleotides. It should be noted that some differences in the formation of indels were observed when editing different exons and different regions of the same exon. Exon 1 is 361 bp in size, and exon 3 is 860 bp. The maximum deletion size in exon 1 was 14 nucleotides, whereas in exon 3 a deletion size reached 54 nucleotides. Among all +1 indel mutations, insertion of C was prevalent in exon 1, whereas insertion of T was prevalent in exon 3. Additionally, indels that did not cause an open reading frame shift (i. e. multiples of three nucleotides) occurred more often in exon 3.

The differences in editing various sites within the same exon were primarily related to the efficiency of indel mutation generation. Knockout efficiency was six times lower with sgRNA-P3.A than with sgRNA-P3.B. Notably, the distance between the Cas9 target sites in exon 3 was only 61 bp. The difference in the knockout efficiency was even greater for guide RNAs sgRNA-P1.A (43.8 %) and sgRNA-P1.B (0 %), which were 137 bp apart.

To visually confirm the inactivation of vacuolar invertase in the Fritella regenerants, an experiment involving frying chips was conducted. Of the 14 regenerants obtained with Vectors A and B, 11 demonstrated the knockout of all gene alleles. When slices of tubers from knockout sample 11 - 2 (Vector A) were fried, the chips exhibited dark coloring with small light segments. This effect may have been due to number of causes. For instance, dark chip color resulting from high hexose levels may have been an individual response of the transformant to altered sucrose metabolism as a consequence of the StPain-1 gene knockout. Accumulation of hexoses in this case was probably caused by another sucrose-hydrolyzing enzyme, such as sucrose synthases or homologous invertases.

Alternatively, as a result of Agrobacterium-mediated transformation, T-DNA may have been inserted into the plant genome, often in euchromatic regions. This genome modification could result in altered expression of certain genes, which could subsequently cause changes in sucrose metabolism. Another cause of the observed effect could be the chimerism of the regenerated plant. The presence of cells with different genotypes within a single plant is a well-known phenomenon that occurs during the Agrobacterium-mediated transformation of callus. A notable example of this effect can be seen in a plant obtained by inactivating the StLEAFY gene (Lebedeva et al., 2022).

Three other regenerants whose tubers were used to make chips retained one or two wild-type alleles (26 - 1, 20 - 1, 50 - 2). However, dark coloration was only observed in the chips from sample 50 - 2. Indeed, a decreased content of reducing sugars had previously been observed when the StPain-1 gene was knocked down (Yasmeen et al., 2022).

Several hypotheses can be proposed to explain this phenomenon. Tetraploid potatoes are known for their high degree of heterozygosity. Different StPain-1 alleles of the gene may contain nucleotide polymorphisms that affect the enzymatic activity of vacuolar invertase (Draffehn et al., 2010; Slugina, Kochieva, 2014). In this regard, inactivation of the allele encoding the most active enzyme can lead to a significant decrease in CIS and vice versa. Also, the reaction rate can be influenced by the amount of enzyme, which is determined by systems that regulate cellular gene expression. Moreover, mRNA synthesis most often occurs in an allele-specific manner (Pham et al., 2017). Therefore, introducing mutations into the intensely transcribed StPain-1 alleles could also affect the glucose and fructose contents in tubers of the transformants under study.

## Conclusion

Plants with increased resistance to CIS were obtained using the genome editing method, based on the Russianbred Fritella potato variety intended for industrial processing. These plants show great promise for breeding new potato varieties and can be used as donors of nonfunctional alleles of the vacuolar invertase gene.

## Conflict of interest

The authors declare no conflict of interest.
